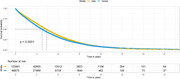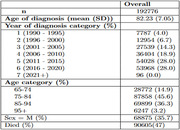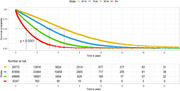# Predicting individual‐level stroke risk associated with risperidone use in dementia using cardiovascular disease history: matched UK population based cohort study

**DOI:** 10.1002/alz.087649

**Published:** 2025-01-09

**Authors:** Nalamotse Joshua Choma, Alys Griffiths, William E Henley, Christoph Mueller, Nefyn Williams, John Dennis, Byron Creese

**Affiliations:** ^1^ University of Exeter, Exeter, Devon United Kingdom; ^2^ University of Sheffield, Sheffield, Sheffield United Kingdom; ^3^ King’s College London, London United Kingdom; ^4^ University of Liverpool, Liverpool United Kingdom; ^5^ Brunel University London, London United Kingdom

## Abstract

**Background:**

Experiencing behavioural symptoms such as aggression, agitation and psychosis contribute significantly to reduced quality of life amongst people with dementia. These behavioral symptoms can be considered more detrimental to overall well‐being than cognitive impairment. In the UK, risperidone is the sole approved atypical antipsychotic for treating these symptoms, despite its notable risk of serious side effects, including stroke. This project aims to develop personalized stroke risk prediction models based on individual clinical features upon introducing risperidone.

**Methods:**

This research will use data from 358,406 patient records contained in the Clinical Practice Research Datalink (CPRD). These data sets include individuals diagnosed with dementia after age 65 after January 1, 1990. The CPRD data will serve as the primary source of information for our study and will facilitate the analysis of stroke risk associated with risperidone use in this specific patient population. Our analysis will focus on two distinct groups, individuals diagnosed with dementia who have been prescribed risperidone and individuals diagnosed with dementia who have never been prescribed risperidone. To achieve our research objectives, we will analyze and compare the incidence of stroke within these two groups. We will then examine whether risk is moderated by individual clinical history. This thorough investigation will form the basis for our predictive models.

**Results:**

Preliminary findings from the data show a well‐defined cohort of dementia cases with an average age of 82 years at diagnosis, consisting predominantly of females (64%). Of the cohort, 87,858 individuals fall into the 75‐84 age category. Over the study period, 47.0% of the cohort died during the study period. The median survival time is approximately 4.4 years for males and 3.7 years for females in the study cohort. This median survival time increases to 6.8 years for people diagnosed at the age of 65‐74 years.

**Conclusions:**

This project’s success could significantly enhance the safety and efficacy of risperidone prescriptions for people with dementia. By leveraging personalized prediction models based on individual clinical features, our research aims to equip clinicians with tools for more informed and patient‐specific decisions concerning risperidone treatment.